# HIV-1-Specific CAR-T Cells With Cell-Intrinsic PD-1 Checkpoint Blockade Enhance Anti-HIV Efficacy *in vivo*

**DOI:** 10.3389/fmicb.2021.684016

**Published:** 2021-07-06

**Authors:** Zhengtao Jiang, Huitong Liang, Hanyu Pan, Yue Liang, Hua Wang, Xinyi Yang, Panpan Lu, Xiao Zhang, Jinlong Yang, Dengji Zhang, Xiaoting Shen, Jing Wang, Zhiming Liang, Qinru Lin, Yanan Wang, Lin Zhao, Yangcheng Zhong, Hongzhou Lu, Huanzhang Zhu

**Affiliations:** ^1^State Key Laboratory of Genetic Engineering and Engineering Research Center of Gene Technology, Ministry of Education, Institute of Genetics, School of Life Sciences, Fudan University, Shanghai, China; ^2^Department of Infectious Disease, Shanghai Public Health Clinical Center, Fudan University, Shanghai, China

**Keywords:** HIV-1 functional cure, cellular immunotherapy, CAR-T, PD-1/PD-L1, immune checkpoint

## Abstract

Adoptive cellular immunotherapy therapy using broadly neutralizing antibody-based chimeric antigen receptor-T cells (bNAb-based CAR-T) has shown great potency and safety for the functional cure of HIV. The efficacy of bNAb-based CAR-T cells could be compromised by adaptive resistance during HIV chronic infection according to the phenomenon that cellular exhaustion was observed in endogenous cytotoxic T-lymphocytes (CTLs) along with upregulated expression of PD−1. Here, we created HIV-specific CAR-T cells using human peripheral blood mononuclear cells (PBMCs) and a 3BNC117-DNR CAR (3BD CAR) construct that enables the expression of PD-1 dominant negative receptor (DNR) and the single-chain variable fragment of the HIV-1-specific broadly neutralizing antibody 3BNC117 to target native HIV envelope glycoprotein (Env). Compared with HIV CAR expression alone, 3BD CAR-T cells displayed potent lytic and functional responses to Env-expressing cell lines and HIV-infected CD4^+^ T cells. Moreover, 3BD CAR-T cells can kill HIV-latently-infected cell lines, which are reactivated by the secretory cytokines of effector cells followed by contact with initial HIV-expressing fraction. Furthermore, bioluminescence imaging indicated that 3BD CAR-T cells displayed superior anti-HIV function in an HIV NCG mouse model of transplanting Env^+^/PD-L1^+^ cells (LEL6). These studies suggested that our proposed combinational strategy of HIV CAR-T therapy with PD-1 blockade therapy is feasible and potent, making it a promising therapeutic candidate for HIV functional cure.

## Introduction

Since the first AIDS case was reported in 1981, more than 38 million people had been infected with HIV worldwide by 2019 [https://aidsinfo.unaids.org/]. Although combination antiretroviral therapy (cART) dramatically suppresses HIV-1 replication, it fails to eliminate the persistent HIV-1 reservoir, which is a major barrier to achieving an HIV cure (Ruelas and Greene, 2013; Siliciano and Siliciano, 2014). The high cost, inconvenience, drug related side effects and shortened life expectancy of the life-long treatment of cART urge us to find a new strategy for functional cure of HIV whereby durable remission is maintained in the absence of continued cART (Archin et al., 2014; Liu et al., 2015).

To suppress the rebound virus for decades after cART termination, potent and persistent cellular immune surveillance are required (Maldini et al., 2018). Adoptive cellular immunotherapy with chimeric antigen receptor (CAR) T cells directed to kill target cell types has achieved great success in the treatment of refractory malignancies and has gradually shown promise against chronic infectious diseases including HIV (Rosenberg and Restifo, 2015). Early attempts at CAR trials for HIV-1 treatment were based on ‘first generation’ CAR constructs, which contained the extracellular domain of CD4 fused to the intracellular signaling domain derived from the CD3ζ chain (Roberts et al., 1994; Tran et al., 1995). The specific lysis of HIV-infected cells can be redirected by the interaction of CD4 molecules on CAR-T cells and HIV-1 Env *in vitro* (Roberts et al., 1994; Tran et al., 1995; Masiero et al., 2005). Although the ‘first generation’ CD4-CAR was safe and CAR^+^ cells were detectable for 10 years (Scholler et al., 2012), it has no durable control of virus in HIV patients (Deeks et al., 2002). The efficacy of this approach was likely compromised because of multiple technical parameters including low transduction efficiencies, absence of intracellular costimulatory signaling domain and the potential for HIV infection of T cells expressing the CD4 CAR (Wagner, 2018). Along with the advances achieved in cancer CAR-T therapy, the efficacy of anti-HIV CAR-T cells could be enhanced based on improved ‘second generation’ and ‘third generation’ CAR_S_ containing additional intracellular costimulatory domains such as CD28 and 4-1BB (Hombach et al., 2013; Maldini et al., 2020; Ollerton et al., 2020).

An alternative approach to using the extracellular region of the CD4 receptor for targeting the HIV Env is a single-chain variable fragment (scFv) derived from bNAbs, which can block cell-cell transmission of HIV-1 and suppress viral replication by recognizing different domains at gp120 and gp41 (Ali et al., 2016; Liu et al., 2016; Hale et al., 2017; Herzig et al., 2019). Over the last decades, various high-affinity bNAbs such as VRC01, VRC07, PGT128, 10E8 and 3BNC117, have been isolated (McCoy and Burton, 2017). Previous studies have shown that 3BNC117-based CAR exhibited potent antiviral activity *in vitro* (Ali et al., 2016). However, one major drawback to developing bNAb-based CARs has been that their functions could be compromised by immunoinhibition. It was reported that specific cytotoxic T lymphocytes (CTLs) in HIV-infected individuals may become exhausted losing their effector function and proliferative capacity over time (Woo et al., 2012), along with upregulated expression of PD-1 (Day et al., 2006; D’Souza et al., 2007), Tim3 (Porichis and Kaufmann, 2011) or other checkpoint blockers in chronic HIV infection during cART (Kalams et al., 1999; Trautmann et al., 2006; Shin and Wherry, 2007). Targeting these immune checkpoints with antibody therapy may decrease the exhaustion state and counteract immune inhibition (Trautmann et al., 2006; Blackburn et al., 2009; Velu et al., 2009; Porichis et al., 2014; Teigler et al., 2017). Moreover, it has been reported that blockade of the PD-1 pathway can effectively enhance the proliferation and secretion of diverse cytokines on HIV-specific CD4^+^ T cells, not just HIV-specific CD8^+^ T cells (Velu et al., 2009; Porichis and Kaufmann, 2012; Porichis et al., 2014; Teigler et al., 2017). However, antibody-mediated checkpoint blockade therapy needs to be repeated because of the short-lived effects and may result in autoimmune responses (Chen et al., 2017). In contrast to antibodies, blockade of the PD-1 pathway through genetic engineering can make modified cells persist with sustainable cytotoxicity and proliferation for a long time and specifically counteract the inhibition by target cells *in vivo* (Muul et al., 2003; Chen et al., 2017). The immune suppressors of PD-1/PD-L1 in M28z CAR-T cells could be blocked by cotransduction of PD-1 dominant negative receptor (DNR), a decoy receptor that lacks the PD-1 transmembrane and intracellular signaling domains (Cherkassky et al., 2016). Consequently, CAR-T cells co-transduced with DNR had enhanced proliferation, cytotoxicity and cytokine secretion compared to individual CAR-T cells (Cherkassky et al., 2016; Chen et al., 2017). We hypothesized that this strategy could be applied to HIV CAR-T therapy to provide CAR T cell–specific checkpoint blockade.

Here, we utilized an enhanced 3BNC117-DNR CAR (3BD CAR) that can saturate PD-1 ligands and thereby block signaling through the endogenous PD-1 receptor. We compared the specific lysing ability of 3BD CAR and individual 3BNC117 CAR (3B CAR) on HIV-1 infected cells. Because of the lack of an appropriate mouse model to study HIV CAR-T therapy (Gardner, 1990; Okuma et al., 2008; Nixon et al., 2017), we constructed a Luc-Env^+^/PD-L1^+^ NCG mouse model that can be continuously monitored by bioluminescence imaging. Subsequently, we assessed the abilities of 3B CAR-T and 3BD CAR-T cells to eliminate HIV target cells in a mouse model. This work might harness individual benefits of CAR T cell therapy and checkpoint blockade therapy to enhance the efficiency of HIV-specific CAR-T cells at targeting HIV-infected cells. Notably, we concluded that this combinatorial immunotherapeutic strategy might be a promising treatment approach for HIV functional cure.

## Results

### Construction of Anti-HIV CARs Derived From 3BNC117 Targeting HIV-1 Envelope Glycoprotein

**FIGURE 1:**
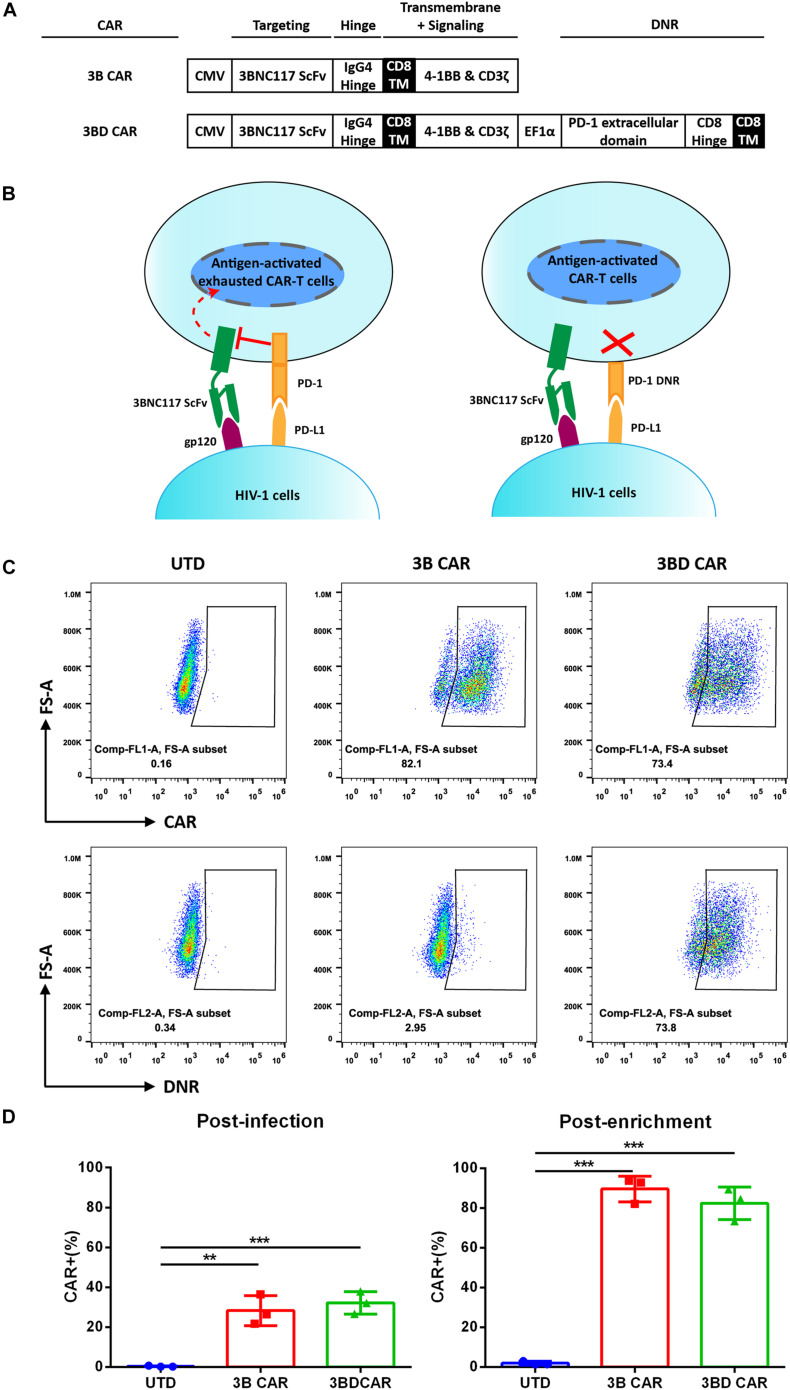
Design and expression of 3BNC117-based CAR constructs. **(A)** Schematic diagram of the 3BNC117 CAR (3B CAR) and 3BNC117-DNR (3BD CAR) constructs. CMV and EF1α indicated in the diagram are promoter sequences. **(B)** Schematic representations of antigen-activated T cells binding PD-L1 via the endogenous PD-1 receptor (transmitting a coinhibitory signal) or DNR lacking an inhibitory signaling domain. **(C)** Representative flow plot showing surface CAR and DNR expression on sorted CD3^+^ T cells by detecting human Fab and CD279 through flow cytometry. Untransduced CD3^+^ T cells (UTD) served as a negative control. **(D)** Percentage of CAR^+^ human primary CD3^+^ cells 4 days after LV transduction (post-infection), and 5 days after enrichment by fluorescence-activated cell sorting (post-enrichment). The data show the mean ± SEM of n = 3 human cell donors. Statistical analysis was performed by one-way ANOVA followed by Tukey’s post-test analysis. **P < 0.01, ***P < 0.001.

The 3BNC117 ScFv gene, which synthesizes of codon-optimized sequences for the heavy and light chains, was linked to ‘second generation’ intracellular moieties containing the 4-1BB signaling domain fused to the CD3ζ signaling domain. The whole composition was cloned into pCDH-CMV-MCS-EF1α-Puro lentiviral (LV) vectors to generate 3BNC117-CAR (3B CAR) (Figure 1A).

It has been reported that the expression of PD-1 on HIV-specific CTLs is upregulated, which results in the exhaustion of HIV-specific CTLs with loss of proliferation and cytolytic functions (Trautmann et al., 2006; Porichis and Kaufmann, 2012; Porichis et al., 2014; Teigler et al., 2017). Therefore, we transduced 3BNC117-CAR with a PD-1 dominant negative receptor (DNR) that contained the extracellular domain of the natural PD-1 molecule fused to a CD8 hinge-transmembrane domain in a lentivirus vector, referring to an attempt at cancer CAR-T therapy (Figure 1A; Cherkassky et al., 2016). The 3BNC117-DNR CAR (3BD CAR) blocked the PD-L1/2 inhibitory signal without any intracellular inhibitory signaling domain (Figure 1B).

CD3^+^ T lymphocytes were isolated and transduced with two anti-HIV CAR LVs or untransduced (UTD) as a control. Flow cytometry demonstrated the expression of CARs on the cell surface using an antibody against human F_ab_. However, only ∼36% of CAR^+^ cells were produced by initial transduction of 3B/3BD CARS (Supplementary Figure 1). To enrich the CAR^+^ cells, transduced cells were stained and sorted for transduced cells by flow cytometry. Five days after sort enrichment, 3BNC117-CAR was efficiently expressed (> 80%) on the T cells surface as shown in Figure 1C. To confirm that DNR was expressed at the 3BD CAR-T cell surface, cells were stained with mouse-anti-human CD279. Although staining with a CD279 antibody was unable to distinguish exogenous PD-1 expression from endogenous PD-1 expression, we found significant differences in PD-1 expression between 3B CAR-T cells (2.95%) and 3BD CAR-T cells (73.8%) (Figure 1C). We found that the expression level of the CAR motif (73.4%) matched that of DNR (73.8%) on 3BD CAR-T cells, as expected. T cells from 3 unique donors were used to produce anti-HIV CAR-T cells using the same method. The expression of CAR was stable at ∼80% to go on to the next experiments (Figure 1D).

### Characterization of Anti-HIV CAR-T Cells Phenotype and Proliferation *in vitro*

**FIGURE 2:**
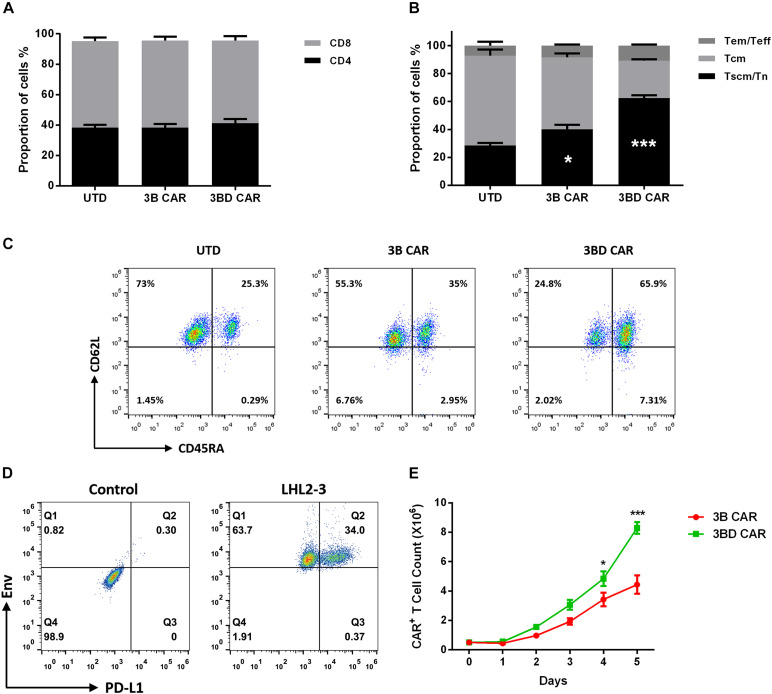
3BD CAR-T cells displayed preferable immunophenotype and proliferation. **(A)** Proportions of CD4^+^ and CD8^+^ T cells in UTD/3B/3BD CAR-T cells. The subset composition of UTD/3B/3BD CAR-T cells were measured by surface expression of CD45RA and CD62L. Shown are summary data **(B)** and representative flow plots **(C)** for indicated CAR groups (combined data from three independent experiments evaluating 3 donors; asterisks indicate significant differences from UTD). T_n_/T_scm_ (CD45RA^+^CD62L^+^), T_cm_ (CD45RA^−^CD62L^+^), T_em_/T_eff_ (CD62L^−^). Data in B was analyzed using two-way ANOVA. **(D)** Flow cytometry was performed to detect the expression of Env and PD-L1 in LHL2/3 cells. **(E)** 3BD CAR-T cells demonstrated enhancement in accumulation. CAR-T cells sorted for CAR expression were incubated with LHL2/3 cells (5 × 10^5^ cells) at 1:1 ratio for 5 days, and CAR^+^ cells were counted daily to evaluate the *in vitro* proliferation. Statistical significance was determined using the unpaired Student’s *t*-test. *p < 0.05, ***P < 0.001. Data represent the mean ± SEM of independent samples.

Prior to functional assay, we measured the phenotype of anti-HIV CAR-T cells. The results showed that the proportions of CD4^+^/CD8^+^ T cells in 3BD CAR was similar to that in UTD/3B CAR (Figure 2A). Importantly, we found a significantly higher percentage of CD45RA^+^/CD62L^+^ double positive cells in the 3BD CAR (Figures 2B,C). These data suggested that there may be more T_n_/T_scm_ cells (CD45RA^+^CD62L^+^) in 3BD CAR, suggesting a potential advantage of 3BD CAR-T cells in persistence and efficacy (Berger et al., 2008; Graef et al., 2014; Ando et al., 2020).

To compare the proliferation of 3B CAR-T cells and 3BD CAR-T cells stimulated by Env antigen, we first constructed a cell line named LHL2/3. LHL2/3, which has PD-L1 overexpression by lentivirus based on HL2/3 (Ciminale et al., 1990) and can constitutively express Env and PD-L1 at the cell surface (Figure 2D). Anti-HIV CAR-T cells were co-cultured with LHL2/3 at 1:1 ratio for 5 days and CAR^+^ cells were counted daily. The data showed that 3BD CAR-T cells had slight but significant advantages in proliferative ability compared with 3B CAR-T cells (Figure 2E).

### DNR Rescues 3BD CAR-T Cells Function on HIV-1 Env^+^ Cells *in vitro*

To compare the capacity of anti-HIV CAR-T cells to kill Env-expressing cells, we first constructed an Env^+^ cell line (LE6) and a luciferase-expressing Env^+^/PD-L1^+^ cell line (LEL6). It was confirmed that 99% of LE6 cells were positive for Env and 92.4% of LEL6 cells were positive for both Env and PD-L1 (Supplementary Figure 2A). To assess whether overexpressed PD-L1 can inhibit function of CAR-T cells, we next tested the cytotoxicity of 3B CAR-T cells in a model of PD-L1–mediated immunoinhibition (LE6 and LEL6). The results suggested that PD-L1 overexpression resulted in decreased lysis upon stimulation in 3B CAR-T cells (Supplementary Figure 2C).

**FIGURE 3:**
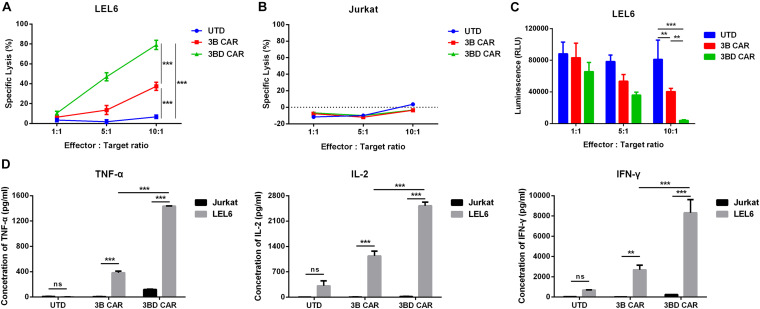
3BD CAR-T cells showed enhanced cytolytic and cytokine secreted function on LEL6 cells by co-transduction of DNR. Anti-HIV CAR-T cells were incubated with target cells at different ratios (1:1; 5:1; and 10:1) for 8 h. **(A)** Direct killing of LEL6 was performed using the LDH release assay. **(B)** Direct cytotoxicity effects on Jurkat cells, as the Env negative control here, were detected. **(C)** Detection of luminescence (RLU) in co-cultures to reflect lysis of LEL6 cells. **(D)** TNF-α, IL-2 and IFN-r production in co-cultures. Anti-HIV CAR-T cells were co-cultured with LEL6 cells (1 × 10^4^ cells) at 10:1 for 24 h, and supernatants were collected for ELISA. Statistical analyses were performed by two-way ANOVA followed by Bonferroni post-test analysis. ns *p* > 0.05, **P < 0.01, ***P < 0.001. Data represent the mean ± SEM of independent samples.

To verify whether 3BD CAR-T cells co-transduced with a genetically engineered PD-1 resistance would provide an advantage in specific cytotoxic activity against Env^+^/PD-L1^+^ cells, the enriched anti-HIV CAR-T cells were then co-cultured with LEL6 cells. The 3B CAR-T cells and 3BD CAR-T cells both displayed robust cytotoxicity against LEL6 at the indicated effector: target (E:T) ratios compared to UTD cells, and increasing ratios led to greater cytotoxic activity on LEL6 cells 8 h post-mixing. Importantly, 3BD CAR-T cells showed greater potency than 3B CAR-T cells at each individual E: T ratio. We found that the killing efficiencies of LEL6 target cells by 3BD CAR-T cells can reached ∼80% at a 10:1 E:T ratio (Figure 3A). In addition, the specificity was demonstrated by the absence of killing activity exhibited in UTD cells and by the lack of cytotoxicity upon Env-negative Jurkat cells (Figure 3B).

Since luciferase was transduced in LEL6 target cells as a reporter gene, we tested the expression level of luciferase after coincubation with anti-HIV CAR-T cells to confirm the killing efficiency further. In addition, 3BD CAR-T cells appeared to show consistent potency in the luciferase assay (Figure 3C). The results showed that although the expression of luciferase could be both reduced by 3B CAR-T cells and 3BC-CAR-T cells at various E:T ratios, the expression of luciferase could barely be detected in 3BD-CAR at a 10:1 E:T ratio (Figure 3C), indicating the potential efficacy of 3BD CAR-T cells in a mouse model.

Anti-HIV CAR-T cells were then tested for their cytokine secretion ability upon interaction with Env-expressing cells. In co-culture with LEL6 at a 10:1 (E:T) ratio, IFN-γ secretion by 3BD CAR-T cells was significantly induced compared with that of 3B CAR-T cells and UTD cells (Figure 3D). Similar results were found for IL-2 and TNF-α release (Figure 3D).

We conclude that 3BD CAR-T cells showed greater potency than 3B CAR-T cells in our initial assays as we expected. We speculate that the specific activation of 3B CAR-T cells was somewhat suppressed by the interaction between PD-1 and PD-L1 on LEL6 cells. These observations suggested the importance of overcoming immune inhibition of CAR-T cells in HIV CAR-T therapy.

### 3BD CAR-T Cells More Effective Against HIV-1-Infected Cells

**FIGURE 4:**
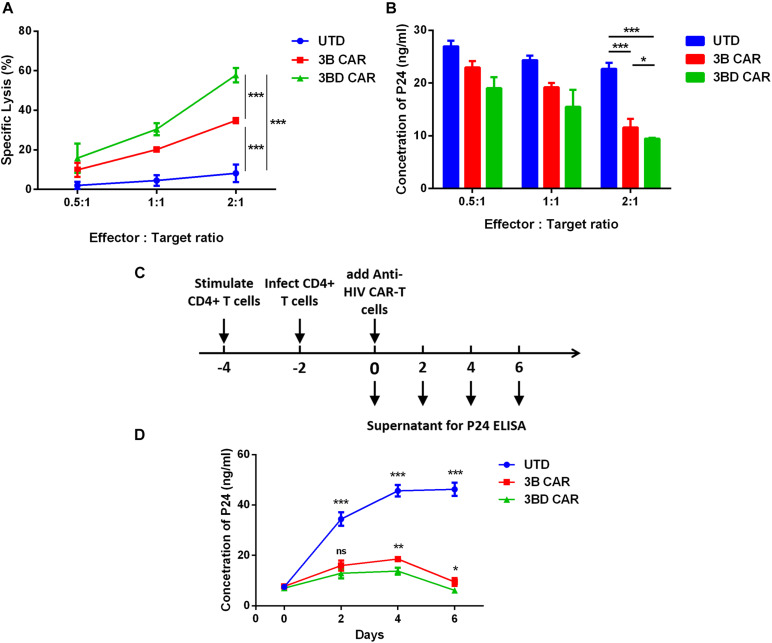
Suppression of spreading HIV-1 infection in CD4^+^ by 3B and 3BD CAR-T cells. **(A)** Primary CD4^+^ T cells were infected with HIV-1_NL4−3_ and mixed with anti-HIV CAR-T cells at different ratios (0.5:1; 1:1; and 2:1). 24 h after co-culture, specific cytotoxicity was measured by LDH assay. **(B)** 48 h after co-culture, the p24 concentration in supernatant was tested. **(C)** To evaluate long-term suppression effects, wild type virus challenge of CAR T cells during a 6-day co-culture with HIV-infected allogenic CD4^+^ T cells at a 2:1 ratio. **(D)** Every two days the cultures were tested for the presence of p24 in the supernatant by ELISA. The significance shown is a comparison of each condition versus 3BD CAR. Statistical analyses were performed using the Tukey method for ANOVA for multiple comparisons. ns *p* > 0.05, *p < 0.05, **P < 0.01, ***P < 0.001. Error bars show ± SEM.

To examine further the effectiveness of anti-HIV CAR-T cells on infected primary cells, CD4^+^ T cells isolated from the same individual were infected with wild-type HIV_NL4–3_ virus. Two days after infection, the cells were co-cultured with anti-HIV CAR-T cells at different ratios from 0.5:1 to 2:1 (E:T) and cytotoxic effects were detected using LDH assay. The data showed that approximately 30% of infected cells were killed by 3B CAR-T cells and that approximately 55% of infected cells were killed by 3BD CAR-T cells at 2:1 ratio (E:T) (Figure 4A). To confirm the antiviral activity of anti-HIV CAR-T cells further, P24 antigen in supernatant was measured to reflect the effects on virus reduction (Figure 4B). These data showed that 3BD CAR-T cells efficiently killed wild-type HIV-infected cells and achieved a significant advance over 3B CAR-T cells.

The functional potency of virus-replication suppression of anti-HIV CAR-T cells was further validated by measurement of p24 antigen between days 0 and 6 of co-culture with infected CD4^+^ T cells (Figure 4C). Through days 0−4, all three groups produced higher levels of virus. However, the increase in 3B/3BD CAR was much lower than that in UTD (Figure 4D). On days 4-6, 3B/3BD CAR-T cells controlled viral replication and there was a significant augmentation of suppression in 3BD CAR-T cells (Figure 4D). These findings support that 3BD CAR-T cells displayed greater potency of antiviral activity than 3B CAR-T cells.

### 3BD CAR-Mediated Specific Killing of HIV Latently Infected Cell Lines

**FIGURE 5:**
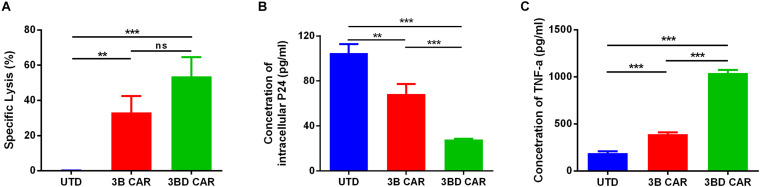
3B and 3BD CAR-T cells kill latently infected cells. ACH-2 cells were mixed with 3B or 3BD CAR-T cells at a 1:1 ratio. **(A)** LDH assay was performed to test specific cytotoxicity at day 1. **(B)** Five days after co-culture, cell pellets were collected, and intracellular P24 was detected by ELISA. **(C)** Production of TNF-α in the supernatant of co-culture at day 1. Statistical analyses were performed using the one-way ANOVA. ns *p* > 0.05, **P < 0.01, ***P < 0.001. Data represent the mean ± SEM.

In prior experiments, we found that Env-expressing cells could be specifically lysed by 3B and 3BD CAR-T cells. We next explored the cytotoxicity of 3B/3BD CAR-T cells on HIV latently infected cells ACH-2, a CEM-based cell line in which constitutive HIV was expressed in only 5% of total cells (Clouse et al., 1989; Folks et al., 1989). ACH-2 cells were co-cultured with 3B CAR-T cells, 3BD CAR-T cells or UTD cells at a 1:1 E:T ratio. LDH assay results showed that 3B CAR-T cells lysed approximately 31% of ACH-2 cells, whereas 3BD CAR-T cells lysed approximately 49% of ACH-2 cells after 1 day of coculture (Figure 5A). After 5 days of co-culture, the P24-ELSA assay results showed that the intracellular P24 concentration in the 3BD CAR was significantly lower than that in the 3B CAR, indicating that more ACH-2 cells were lysed in the 3BD CAR group (Figure 5B). To determine what caused latent ACH-2 cells to reactivate and made them targets, we performed a TNF-α ELISA assay according to previously reported study (Sahu et al., 2013). We found that the concentration of released TNF-α in 3BD CAR-T cells was significantly higher than that in 3B CAR-T cells (Figure 5C).

We found that there was almost no PD-L1 expression in ACH-2 cells (Supplementary Figure 3), which means that the killing activity of anti-HIV CAR-T cells could not be directly affected through the PD-1/PD-L1 pathway, therefore, the difference in specific killing activity at day 1 between 3B CAR and 3BD CAR was not significant (Figure 5).

### 3BD CAR-T Cells Displayed Superior Anti-HIV Function in an HIV NCG Mouse Model

**FIGURE 6:**
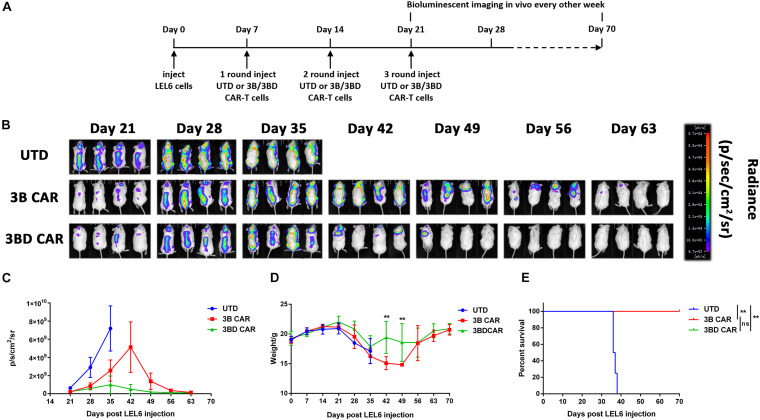
3BD CAR-T cells are more potent than 3B CAR-T cells in eliminating LEL6 cells *in vivo*. **(A)** Illustration of the experimental design. An HIV-1 Luc-Env^+^/PD-L1^+^ NCG mouse model was established in NCG mice by inoculating 1 × 10^5^ LEL6 cells/mouse (i.v. by tail, *n* = 4). In the next 3 weeks, the mice were treated with 3B CAR-T cells or 3BD CAR-T cells 3 times. Mice treated with non-transduced T cells (UTD) served as the control. BLI was conducted after the mice were treated with the last single T cell injection (Day 21) and performed weekly. **(B)** LEL6 progression and distribution were evaluated by serial bioluminescence imaging. **(C)** Bioluminescence values of mice receiving different treatments are displayed. **(D)** Sequential body weight was analyzed every 7 days after LEL6 cell implantation. The significance shown is a comparison of each condition versus 3B CAR. Statistical analyses were performed by two-way ANOVA followed by Bonferroni post-test analysis. *p < 0.05, **P < 0.01. **(E)** The survival of the mice was monitored. (**p < 0.01; log rank).

The efficiency of anti-HIV CAR-T cell killing of Env-expressing cells was preliminarily proven in our initial assays *in vitro*. To study whether administration of 3BD CAR-T cells co-transduced with DNR would provide an *in vivo* advantage, NCG mice were intravenously (i.v.) engrafted with 1 × 10^5^ LEL6 cells followed by injection of 3B/3BD CAR-T cells (*n* = 4). Control mice were injected with UTD cells. Subsequently, a bioluminescence imaging system was used to observe the signal of LEL6 cells, representing HIV/AIDS progression, every week *in vivo* (Figure 6A). Engraftment and growth of LEL6 cells was evident in all mice by day 21 and increased significantly in the UTD/3B CAR group by days 35∼42 (Figure 6B). Treatment with 3B CAR-T cells exhibited limited efficacy of decreased luciferase-expression by days 42∼56 and had complete LEL6 elimination on day 63 (Figures 6B,C). In contrast, mice treated with 3BD CAR-T cells showed greater extinction of luciferase expression than 3B CAR-T cells by days 42∼56 and remained LEL6-free by days 49∼63 (Figures 6B,C). These results demonstrated that 3BD CAR-T cells were more effective in eliminating HIV Env + cells in NSG mice than 3B CAR-T cells.

Moreover, the contribution of anti-HIV CAR-T cells to mouse survival was assessed. We found decreased weight loss rates in 3BD CAR-treated mice compared to 3B CAR-treated or UTD-treated mice by monitoring weight (Figure 6D). None of the UTD-treated mice survived 35 days post transplanting, whereas 3B/3BD CAR-treated mice survived for the duration of the experiments (Figure 6E).

Taken together, the results show that 3BD CAR-T cells are more potent in the anti-HIV response than 3B CAR-T cells *in vivo*. Our data also provided an aggressive HIV-1 mouse model, which is operational and easy to monitor with a bioluminescence imaging system, for HIV-1 adoptive cellular immunotherapy that uses envelope glycoprotein or PD-1/PD-L1 as targets.

## Discussion

Currently, the major barriers to an HIV cure are the HIV latent reservoir and high viral mutation rates (Goulder et al., 1997; Chapuis et al., 2011). To build up a long-term anti-HIV-1 immune surveillance for functional cure of HIV, adoptive cellular immunotherapy therapy using CAR-T cells should be addressed (Katlama et al., 2013; Kuhlmann et al., 2018; Liu et al., 2019). Many attempts of bNAb-based CAR-T therapies have proven effective *in vitro* as previously reported (Ali et al., 2016; Liu et al., 2016; Hale et al., 2017; Herzig et al., 2019). However, the risk of anti-HIV CAR-T cell immune inhibition has not been taken into account.

Our goal is to generate enhanced anti-HIV CAR-T cells that not only are highly potent and specific, but also avoid immune inhibition. In view of concerns that PD-1 plays an important role in mediating T cell exhaustion during HIV chronic infection (Petrovas et al., 2006; Porichis and Kaufmann, 2012), our approach to overcome inhibition of anti-HIV CAR-T cells is using DNR to intervene in the PD-1/PD-L1 pathway. To investigate whether overexpression of DNR will enhance anti-HIV CAR-T immune responses, we first constructed a Jurkat-based cell line that expresses Env from HIV-1 isolates HXB2 and PD-L1 at the cell surface (Supplementary Figure 2A). We found that both 3B CAR-T cells and 3BD CAR-T cells could effectively lyse Env^+^/PD-L1^+^ (LEL6) cells and that 3BD CAR-T cells displayed augmented proliferation, killing and cytokine release compared to 3B CAR-T cells (Figures 2B, 3A,D). More importantly, the cytotoxic data implied that wild-type HIV-1-infected CD4^+^ T lymphocytes could be lysed by 3B/3BD CAR-T cells, consistent with the supernatant P24 assay (Figure 4). In summary, these studies identified that overexpressing DNR to intervene in the PD-1/PD-L1 pathway was better than the individual 3BNC117-CAR.

Although it has been previously reported that bNAb-based CAR-T cells can effectively kill the reactivated CD4^+^ cells from HIV-1-infected individuals on ART *in vitro* (Liu et al., 2016; Herzig et al., 2019), it was uncertain whether bNAb-based CAR-T cells could kill latently infected cells without latency-reversing agents (LRAs). Therefore, we co-cultured anti-HIV CAR-T cells with ACH-2 cells to investigate its potency on latently infected cells. Unexpectedly, approximately 50% of ACH-2 cells were lysed by 3BD CAR-T cells instead of the small 5% HIV-expressing fraction (Figure 5A). The advantage of 3BD CAR-T cells was further demonstrated by detection of intracellular P24 level (Figure 5B). This outcome is most likely attributed to TNF-α released from 3BD CAR-T cells in co-culture supernatants causing the reactivation of latent HIV in ACH-2 cells, leading to their lysis by 3BD CAR-T cells (Figure 5C; Sahu et al., 2013). These data suggested the potency of 3BD CAR-T cells with two functions: activating HIV-1 latently infected cells and killing reactivated cells. Thus, there could be a potential efficacy in the treatment of HIV individuals on ART by grafting 3BD CAR-T cells, which results in the targeting of persistently active HIV reservoirs (Poles et al., 2006; Minang et al., 2010). However, further tests are needed before its application in the clinic.

A major finding of our study is the further corroboration that 3B/3BD CAR-T cells have the ability to eliminate Env^+^ cells *in vivo* using a Luc-Env^+^/PD-L1^+^ NCG mouse model. Mice treated with 3BD CAR-T cells had significantly enhanced HIV Env^+^ cell control and decreased weight-loss-rates (Figure 6B-D). The results of improved survival and weight rates further indicated the potent anti-HIV activity of 3BD CAR-T cells *in vivo* (Figures 6D,E). The use of this *in vivo* model provides proof that it is a feasible and aggressive HIV mouse model in preliminary investigation of HIV immunotherapy focusing on targeting envelope glycoprotein or the PD-1/PD-L1 pathway, compared to BLT mice, hu-spl-PBMC-NSG mice or other models used in previous research (Anthony-Gonda et al., 2019; Maldini et al., 2020). The results *in vivo* also support 3BD CAR-T cells as a potential strategy to eliminate Env-expressing cells.

Our studies also indicate that a combinatorial approach of HIV-1 CAR-T therapy and immune checkpoint therapy is a potential strategy toward HIV functional cure. In addition to PD-1, other immune checkpoint molecules, such as Tim3 and CTLA-4, were upregulated on HIV-infected patients’ T cells, and we can also explore whether combination of anti-HIV CAR-T therapy with blocking these pathways could increase the HIV-specific immune response in the future (Tian et al., 2015; Fromentin et al., 2016). Furthermore, anti-HIV CAR-T cells can be genetically modified with co-expression of secreted ScFvs targeting the immune checkpoint, especially referring to the design of CD19-CAR-T cells secreting PD-1-blocking ScFv in tumor CAR-T therapy (Rafiq et al., 2018). The anti-HIV CAR-T cells modified through this approach will not only interfere with HIV-1 latency by PD-1-blocking ScFv (Said et al., 2010; Hentrich et al., 2017), but also effectively enhance the ability of CAR-T cells to kill reactivated HIV-1 infected cells to achieve “shock and kill” with one operation.

In summary, we have demonstrated the feasibility and potency of a novel approach for anti-HIV CAR-T therapy through rational combination of anti-HIV CAR-T therapy and PD-1 checkpoint blockade. Moreover, such a therapeutic strategy could probably eliminate reactivated HIV latently infected cells in HIV-infected individuals, supported by the robust efficiency of 3BD CAR-T cells in a Luc-Env^+^/PD-L1^+^ NCG mouse model. Ultimately, our data strongly indicated that 3BD CAR-T cells are powerful therapeutic candidates to provide effective immune surveillance on HIV after ART interruption and warrant further studies of combination with other approaches to achieve a functional cure for HIV-1.

## Materials and Methods

### Ethics Statement

This study was approved by the Ethics Committee of Shanghai Public Health Clinical Center and the methods were consistent with the relevant guidelines and regulations of that committee. All immunodeficiency mouse experiments were approved by the University of Fudan’s Institutional Animal Care and Use Committee. The experiments were carried out in accordance with recommendations in the Guide for the Care and Use of Laboratory Animals of Fudan University.

### Construction of Lentiviral Vectors Encoding Anti-HIV CARS

The plasmid containing sequences of 3BNC117-ScFv, IgG4 Hinge, human CD8 transmembrane sequence, cytoplasmic domains of human 4.1BB (CD137) and human CD3 complex ζ chain (CD247) was provided as the generous gift of Dr. Otto O. Yang (Ali et al., 2016). The backbone for novel CAR constructs (pCDH-CMV-MCS-EF1-Puro) was purchased from Youbio (Youbio, shanghai, China). The HIV-1 HXB2 Env expression vector (pIIIenv3-1) was obtained from Dr. Joseph Sodroski (cat# 289) through the NIH AIDS Reagent Program (Sodroski et al., 1986). The Env and luciferase genes were cloned into the lentiviral backbone pCDH with puromycin resistance gene. The PD-L1 gene (NC_000009.12) was synthesized by GENEWIZ (GENEWIZ, Suzhou, China) and cloned into the lentiviral backbone pCDH with the hygromycin resistance gene.

To obtain 3BNC117-pCDH CAR (3B CAR), the CAR moiety was inserted into the *Eco*RI-*Sal*I restriction fragment into the lentiviral vector pCDH. For construction of 3BNC117-DNR-pCDH (3BD CAR), the 3BNC117 CAR moiety was inserted into the *Eco*RI-*Bam*HI restriction fragment. DNR, which synthesized by GENEWIZ to encode the extracellular portion of the PD-1 receptor fused to the CD8 transmembrane and hinge domains, was inserted into the *Xma*I-*Sal*I restriction fragment into the lentiviral vector mentioned above.

### Cell Lines

As previously mentioned, HL2/3 cells (obtained through the NIH AIDS Reagent Program, Division of AIDS, NIAID, NIH: HL2/3 from Dr. Barbara K. Felber and Dr. George N. Pavlakis) express high levels of HIV-1 proteins but no infectious virus (Ciminale et al., 1990). To generate LHL2/3 cells, HL2/3 cells were transduced with lentivirus containing the PD-L1 gene and selected for positive clones with Hygromycin B (Yeason, Shanghai, China).

HEK293T cells purchased from ATCC (Manassas, VA, United States) were cultured in DMEM supplied with 10% (v/v) fetal bovine serum (FBS) (Gibco, Grand Island, NY, United States) and 1% penicillin-streptomycin (Gibco).

The Jurkat cell line was purchased from ATCC and used as an envelope-negative cell line. To generate target cells that were used for Env^+^ cytotoxicity assays, Jurkat cells were modified to express, constitutively, high levels of the gp160 envelope derived from HXB2, PD-L1 and luciferase and designated LEL6. Briefly, Jurkat cells were transduced with a lentiviral vector containing the gp160 gene and firefly luciferase gene. Then, a single cell clone (LE6) that expressed a high level of luciferase was isolated and transduced with lentivirus to overexpress PD-L1. HIV-1 Env^+^/PD-L1^+^ Jurkat cells (LEL6) were obtained with 50 μg/ml Hygromycin B and subsequently tested for Env/PD-L1 expression on the surface. ACH-2 cells were obtained through the NIH AIDS Reagent Program, Division of AIDS, NIAID, NIH: ACH-2 Cells from Dr. Thomas Folks (cat# 349) (Clouse et al., 1989; Folks et al., 1989).

### Pseudoviruses Production

HEK293T cells were seeded at 8 × 10^6^ cells per 10-cm dish. 16 h later, pseudoviruses were generated by co-transfecting HEK293T cells with plasmids pCDH encoding various CAR moieties (10 μg), Δ8.91 (8 μg) and VSVG (6 μg) using PEI following the manufacturer’s instructions. Viral supernatant was collected 48 h and 72 h after the media change. Cell debris was removed by centrifugation at 7000 rpm for 20 min, followed by filtration through a 0.45 μM membrane (Millipore, Boston, MA, United States). Pseudoviruses were then concentrated by centrifugation at 25000 rpm for 2 h at 4°C. The Pellets were resuspended in serum-free vivo 15 and stored in −80°C.

Titration of lentiviral vectors was detected by quantitative PCR to determine the number of vector copies associated with genomic DNA extracted from transduced 293T cells as previously reported (Kutner et al., 2009). Briefly, 293T cells were transduced with lentivirus stock and genomic DNA from transduced 293T cells were collected for qPCR. qPCR was performed with QuantiFast^®^ SYBR^®^ Green PCR kit (QIAGEN, Düsseldorf, Germany) using Roche LightCycler 480 II PCR system (Roche, Basel, Switzerland). The primer sequences used are as follows: WPRE-F: GGCACTGACAATTCCGTGGT; WPRE-R: AGGGACGTAGCAGAAGGACG; glyceraldehyde-3-phosphate dehydrogenase (GAPDH)-F: GGACAGGACCATATTGAGGGACA; GAPDH-R: AGGAGTGAGTGGAAGACAGAATGGA.

### Human T Lymphocyte Isolation and Generation of Anti-HIV CAR-T Cells

White blood cells from healthy donors were obtained from the Blood Center of Shanghai (Shanghai, China). Human PBMCs were isolated from whole blood by Ficoll-Paque gradient separation (GE Healthcare, Boston, MA, United States). CD3^+^ T cells were enriched by negative immunomagnetic bead selection according to the manufacturer’s protocol (Miltenyi Biotec, Germany). Isolated CD3^+^ T cells were stimulated with CD3/CD28 T-cell activation Dynabeads (Gibco) for 48 h. For infection, 1 × 10^6^ stimulated CD3^+^ cells were transduced with concentrated pseudovirus supernatant (MOI = 10) plus 7 μg/ml polybrene (Yeason) for 12 h, and then, the second round of infection was carried out with the same procedure. T cells were expanded in complete T cell medium containing 90% vivo15 (Lonza, Basel, Switzerland) supplemented with 10% FBS and 100 U/ml penicillin/streptomycin. Cells were also fed with 5 ng/ml IL-2 (R&D, St. Paul, MN, United States), 2 ng/ml IL-7 (R&D) and 2 ng/ml IL-15 (R&D) every 48 h. 4-7 days after LV transduction, CAR^+^ cells were enriched by staining with FITC-conjugated goat anti-human F(ab’)_2_ antibody (Jackson ImmunoResearch Laboratories, West Grove, United States) and sorted by a FACS Fusion I (BD Biosciences, Grand Island, NY, United States). Genetically modified T cells were used for functional assays after sorting.

### Flow Cytometry

For surface staining of the CAR moiety, FITC-conjugated goat anti-human F(ab’)_2_ antibody was used. PE-conjugated mouse anti-human CD279 (PD-1) Antibody (clone EH12.2H7, BioLegend, Santiago, CA, United States) was used for DNR staining. For LEL6 cells, Env was stained by Biotin-Goat Anti-Human gp120 (Abcam, Cambridge, Cambridgeshire), followed by APC Streptavidin (BD Biosciences). PD-L1 was stained with PE Mouse Anti-Human CD274 (clone MIH1, BD Biosciences). The percentage of CD4^+^ and CD8^+^ T cells were determined by flow cytometry with staining of FITC Mouse Anti-Human CD4 (clone RPA_T4, BD Biosciences) and APC Mouse Anti-Human CD8 (clone RPA_T8, BD Biosciences). To analysis the phenotype of CAR-T cells, the expression of CD62L, CD45RA were determined by flow cytometry with staining of FITC Mouse Anti-Human CD45RA (clone HI100, BD Biosciences) and PE Mouse Anti-Human CD62L (clone DREG_56, BD Biosciences). All modified cells were washed, resuspended in 100 μl of PBS containing individual antibodies and incubated for 30 min at 4°C. Cells were then washed and harvested in PBS. Data were acquired on a Beckman Coulter Gallios flow cytometer and were analyzed with FlowJo software (Tree Star, Ashland, OR).

### Proliferation Assay

To analyze the proliferation, CAR^+^ T cells were stimulated on LHL2/3 cells at a 1:1 E:T ratio in triplicate. T cell numbers were counted daily, with plotted numbers adjusted for CAR^+^ percentage as determined by flow cytometry, using the Countess II (Thermo Fisher Scientific, MA, United States).

### Cytotoxicity Assay

The specific killing activity of anti-HIV CAR-T cells toward Jurkat cells, LE6 cells, LEL6 cells, ACH-2 cells or HIV-1 infected primary CD4^+^ T lymphocytes at different ratios from 10:1 to 0.5:1 was measured by lactate dehydrogenase assay (LDH assay) using the CytoTox 96 non-radioactive cytotoxicity kit (Promega, Madison, WI, United States). The manufacturer’s instructions were followed. Briefly, 1 × 10^4^ target cells were co-cultured with anti-HIV CAR-T cells at various E:T ratios in a 96-well U-bottom plate and incubated for 8-24 h at 37°C. The supernatants were collected and incubated with 50 μL Cytotox 96 regent for 30 min. 50 μL Stop solution were added to each well followed by quantification of absorbance at 490 nm.

The cytotoxic effects of anti-HIV CAR-T cells on LEL6 cells were also confirmed by luminescence detection. CAR-T cells were co-culture with LEL6 cells (1 × 10^4^ cells) at different ratios from 10:1 to 1:1, 8 h later, 100 μL of Glo reagent (Promega) was added to each well and incubated for 10 min. Then, luciferase activity was measured by an inspired plate reader (Biotek, Montpelier, VT, United States), reflecting the cytotoxicity effects.

### Enzyme-Linked Immunosorbent Assay (ELISA) Assay

24 h after co-culture of CAR-T cells and LEL6 cells, supernatants were collected for IFN-γ (DAKEWE, Shenzheng, China), TNF-a (DAKEWE), and IL-2 (DAKEWE) ELISA assays. HIV-1 viral particles were monitored using a P24 ELISA kit (R&D). All assays were performed according to the manufacturers’ instructions.

### *In vitro* Wild-Type HIV Infection and Virus Suppression Assays

CD4^+^ T cells were enriched by negative immunomagnetic bead selection according to the manufacturer’s protocol (Miltenyi Biotec) followed by stimulation with CD3/28 beads for 48 h. On day 0, 1 × 10^6^ stimulated CD4^+^ T cells were infected with 1 μl HIV_NL4–3_ (P24 titer of 1 ng/mL) for 4 h. 24 h after media change, infected CD4^+^ T cells (2 × 10^4^ cells) were co-cultured with CAR-T cells at different ratios from 1:0.5 to 1:2. The cytotoxicity was measured by LDH assay at Day 1, and the co-cultured supernatants were collected at Day 2 for p24 detection. To evaluate long-term suppression effects, anti-HIV CAR-T cells were co-cultured with infected CD4^+^ T cells at a 2:1 ratio. Supernatants were collected from wells, replaced with an equivalent volume of fresh medium every 48 h for the next 6 days and p24 concentrations were quantified by ELISA kit (R&D).

### *In vivo* CAR-T Efficacy Using a Luc-Env^+^/PD-L1^+^ NCG Mouse Model

Five week old NCG (NOD/ShiLtJGpt-*Prkdc^*em*26*Cd*52^Il2rg^*em*26*Cd*22^*/Gpt) mice were obtained from GemPharmatech (Nanjing, China), and at 6 weeks, they were injected intraperitoneally (i.p.) with 1 × 10^5^ LEL6 cells. 7 days later, the mice were injected with 1 million UTD cells/3B CAR-T cells/3BD CAR-T cells. The second-round and third-round injections of 2 million UTD cells/3B CAR-T cells/3BD CAR-T cells were performed at Day 14 and Day 21 post LEL6 cell injection.

Mice were subjected to weekly bioluminescence imaging. Briefly, mice were anesthetized and injected intraperitoneally (i.p.) with luciferin substrate (150 mg/kg) (Perkin Elmer, Waltham, MA, United States). Luciferase activity was measured within 10 min using NightOW LB983 (Berthold). Data were analyzed and exported using IndiGO (Berthold, Stuttgart, Germany). Luminescence signal intensity is represented by radiance in photons per second per centimeter squared per steradian (p/sec/cm^2^/sr). Mice were monitored weekly for weight loss and mortality for 70 days. Normally, we assumed that mice die by default when their weight drops by 25%.

### Statistical Analysis

Statistical analyses were performed using GraphPad Prism 6 (GraphPad, San Diego, CA, United States). Tests of statistical significance were performed using unpaired two-tailed Student’s *t* test, and one-way analysis of variance (ANOVA) or two-way ANOVA was used for comparison among multiple groups. Differences were considered statistically significant when *P* < 0.05. Survival curves were prepared via the product-limit method of Kaplan and Meier, and comparisons were analyzed with using the log-rank test.

## Data Availability Statement

The original contributions presented in the study are included in the article/Supplementary Material, further inquiries can be directed to the corresponding author/s.

## Ethics Statement

The studies involving human participants were reviewed and approved by Ethics Committee of Shanghai Public Health Clinical Center. The patients/participants provided their written informed consent to participate in this study. The animal study was reviewed and approved by University of Fudan’s Institutional Animal Care and Use Committee.

## Author Contributions

HZ was responsible for conceiving and designing of the whole study. ZJ performed most experiments and analyzed the data. HL, YL, PL, HP, XZ, XY, JY, XS, JW, ZL, YW, LZ, and QL participated in some of the experiments. HW, DZ, YZ, and HL kindly provided some suggestions for some experiences and revised the manuscript. The manuscript was prepared by ZJ and HZ. All authors read and approved the submitted manuscript.

## Conflict of Interest

The authors declare that the research was conducted in the absence of any commercial or financial relationships that could be construed as a potential conflict of interest.

## Supplementary Material

Supplementary Figure 1 Detection of CARs on transduced CD3 T cells before sorting. The 3BNC117 CAR was detected with FITC-labeled goat anti-human IgG antibody; untransduced CD3^+^ T cells (UTD) served as a negative control.

Supplementary Figure 2 Generation of Env-expressing target cells. **(A)** Detection of Env and PD-L1 on LE6/LEL6 cells. Env was detected with a biotin-labeled goat anti-human gp120 antibody and APC streptavidin. PD-L1 was detected with a PE-labeled mouse anti-human CD274 antibody. Jurkat cells served as a negative control. **(B)** Detection of luminescence (RLU) on different numbers of LEL6 cells with a Luc assay. **(C)** PD-L1 inhibits 3B CAR-T cells cytolytic function. 3B CAR-T cells sorted for CAR expression were incubated with LE6 or LEL6 respectively at 10:1 (E: T) ratio for 8 h, and then the direct killing was detected by LDH assay. UTD served as a negative control. Statistical analyses were performed by two-way ANOVA followed by Bonferroni post-test analysis. ^∗∗^*P* < 0.01. Data represent the mean ± SEM.

Supplementary Figure 3 Flow cytometry for the detection of PD-L1 in ACH-2 cells. PE-labeled mouse anti-human CD274 antibody was used to determine the expression of PD-L1 on ACH-2 cells.

## References

[B1] Ali A. Kitchen S. G. I Chen S. Y. Ng H. L. Zack J. A. Yang O. O. (2016). HIV-1-specific chimeric antigen receptors based on broadly neutralizing antibodies. *J. Virol.* 90 6999–7006. 10.1128/jvi.00805-16 27226366 PMC4944295

[B2] Ando M. Ito M. Srirat T. Kondo T. Yoshimura A. (2020). Memory T cell, exhaustion, and tumor immunity. *Immunol. Med.* 43 1–9. 10.1080/25785826.2019.1698261 31822213

[B3] Anthony-Gonda K. Bardhi A. Ray A. Flerin N. Li M. Chen W. (2019). Multispecific anti-HIV duoCAR-T cells display broad in vitro antiviral activity and potent in vivo elimination of HIV-infected cells in a humanized mouse model. *Sci. Transl. Med.* 11:eaav5685. 10.1126/scitranslmed.aav5685 31391322 PMC7136029

[B4] Archin N. M. Sung J. M. Garrido C. Soriano-Sarabia N. Margolis D. M. (2014). Eradicating HIV-1 infection: seeking to clear a persistent pathogen. *Nat. Rev. Microbiol.* 12 750–764. 10.1038/nrmicro3352 25402363 PMC4383747

[B5] Berger C. Jensen M. C. Lansdorp P. M. Gough M. Elliott C. Riddell S. R. (2008). Adoptive transfer of effector CD8+ T cells derived from central memory cells establishes persistent T cell memory in primates. *J. Clin. Invest.* 118 294–305. 10.1172/jci32103 18060041 PMC2104476

[B6] Blackburn S. D. Shin H. Haining W. N. Zou T. Workman C. J. Polley A. (2009). Coregulation of CD8(+) T cell exhaustion by multiple inhibitory receptors during chronic viral infection. *Nat. Immunol.* 10 29–37. 10.1038/ni.1679 19043418 PMC2605166

[B7] Chapuis A. G. Casper C. Kuntz S. Zhu J. Tjernlund A. Diem K. (2011). HIV-specific CD8+ T cells from HIV+ individuals receiving HAART can be expanded ex vivo to augment systemic and mucosal immunity in vivo. *Blood* 117 5391–5402. 10.1182/blood-2010-11-320226 21422474 PMC3109713

[B8] Chen N. Morello A. Tano Z. Adusumilli P. S. (2017). CAR T-cell intrinsic PD-1 checkpoint blockade: a two-in-one approach for solid tumor immunotherapy. *Oncoimmunology* 6:e1273302. 10.1080/2162402x.2016.1273302 28344886 PMC5353939

[B9] Cherkassky L. Morello A. Villena-Vargas J. Feng Y. Dimitrov D. S. Jones D. R. (2016). Human CAR T cells with cell-intrinsic PD-1 checkpoint blockade resist tumor-mediated inhibition. *J. Clin. Invest.* 126 3130–3144. 10.1172/jci83092 27454297 PMC4966328

[B10] Ciminale V. Felber B. K. Campbell M. Pavlakis G. N. (1990). A Bioassay for Hiv-1 based on Env-Cd4 Interaction. *AIDS Res. Hum. Retroviruses* 6 1281–1287. 10.1089/aid.1990.6.1281 2078409

[B11] Clouse K. A. Powell D. Washington I. Poli G. Strebel K. Farrar W. (1989). Monokine regulation of human immunodeficiency virus-1 expression in a chronically infected human T cell clone. *J. Immunol.* 142 431–438. 2463307

[B12] Day C. L. Kaufmann D. E. Kiepiela P. Brown J. A. Moodley E. S. Reddy S. (2006). PD-1 expression on HIV-specific T cells is associated with T-cell exhaustion and disease progression. *Nature* 443 350–354. 16921384 10.1038/nature05115

[B13] Deeks S. G. Wagner B. Anton P. A. Mitsuyasu R. T. Scadden D. T. Huang C. (2002). A phase II randomized study of HIV-specific T-cell gene therapy in subjects with undetectable plasma viremia on combination antiretroviral therapy. *Mol. Ther.* 5 788–797. 10.1006/mthe.2002.0611 12027564

[B14] D’Souza M. Fontenot A. P. Mack D. G. Lozupone C. Dillon S. Meditz A. (2007). Programmed death 1 expression on HIV-specific CD4+ T cells is driven by viral replication and associated with T cell dysfunction. *J. Immunol.* 179 1979–1987. 10.4049/jimmunol.179.3.1979 17641065

[B15] Folks T. M. Clouse K. A. Justement J. Rabson A. Duh E. Kehrl J. H. (1989). Tumor necrosis factor alpha induces expression of human immunodeficiency virus in a chronically infected T-cell clone. *Proc. Natl. Acad. Sci. U. S. A.* 86 2365–2368. 10.1073/pnas.86.7.2365 2784570 PMC286913

[B16] Fromentin R. Bakeman W. Lawani M. B. Khoury G. Hartogensis W. DaFonseca S. (2016). CD4+ T Cells Expressing PD-1, TIGIT and LAG-3 contribute to HIV persistence during ART. *PLoS Pathog.* 12:e1005761. 10.1371/journal.ppat.1005761 27415008 PMC4944956

[B17] Gardner M. B. (1990). Animal models for development of an AIDS vaccine. *Int. Rev. Immunol.* 7 31–49. 10.3109/08830189009061763 2132878

[B18] Goulder P. J. R. Phillips R. E. Colbert R. A. McAdam S. Ogg G. Nowak M. A. (1997). Late escape from an immunodominant cytotoxic T-lymphocyte response associated with progression to AIDS. *Nat. Med.* 3 212–217. 10.1038/nm0297-212 9018241

[B19] Graef P. Buchholz V. R. Stemberger C. Flossdorf M. Henkel L. Schiemann M. (2014). Serial transfer of single-cell-derived immunocompetence reveals stemness of CD8(+) central memory T cells. *Immunity* 41 116–126. 10.1016/j.immuni.2014.05.018 25035956

[B20] Hale M. Mesojednik T. Romano Ibarra G. S. Sahni J. Bernard A. Sommer K. (2017). Engineering HIV-resistant, Anti-HIV chimeric antigen receptor T Cells. *Mol. Ther.* 25 570–579. 10.1016/j.ymthe.2016.12.023 28143740 PMC5363191

[B21] Hentrich M. Schipek-Voigt K. Jager H. Schulz S. Schmid P. Stotzer O. (2017). Nivolumab in HIV-related non-small-cell lung cancer. *Ann. Oncol.* 28 2890–2890. 10.1093/annonc/mdx321 29106466

[B22] Herzig E. Kim K. C. Packard T. A. Vardi N. Schwarzer R. Gramatica A. (2019). Attacking latent HIV with convertibleCAR-T cells, a highly adaptable killing platform. *Cell* 179 880–894.e10. 31668804 10.1016/j.cell.2019.10.002 PMC6922308

[B23] Hombach A. A. Holzinger A. Abken H. (2013). The weal and woe of costimulation in the adoptive therapy of cancer with chimeric antigen receptor (CAR)-redirected T cells. *Curr. Mol. Med.* 13 1079–1088. 10.2174/1566524011313070003 23116267

[B24] Kalams S. A. Goulder P. J. Shea A. K. Jones N. G. Trocha A. K. Ogg G. S. (1999). Levels of human immunodeficiency virus type 1-specific cytotoxic T-lymphocyte effector and memory responses decline after suppression of viremia with highly active antiretroviral therapy. *J. Virol.* 73 6721–6728. 10.1128/jvi.73.8.6721-6728.1999 10400770 PMC112757

[B25] Katlama C. Deeks S. G. Autran B. Martinez-Picado J. van Lunzen J. Rouzioux C. (2013). Barriers to a cure for HIV: new ways to target and eradicate HIV-1 reservoirs. *Lancet* 381 2109–2117. 10.1016/s0140-6736(13)60104-x 23541541 PMC3815451

[B26] Kuhlmann A. S. Peterson C. W. Kiem H. P. (2018). Chimeric antigen receptor T-cell approaches to HIV cure. *Curr. Opin. HIV AIDS* 13 446–453. 10.1097/coh.0000000000000485 29878913 PMC6993924

[B27] Kutner R. H. Zhang X. Y. Reiser J. (2009). Production, concentration and titration of pseudotyped HIV-1-based lentiviral vectors. *Nat. Protoc.* 4 495–505. 10.1038/nprot.2009.22 19300443

[B28] Liu B. Zhang W. Zhang H. (2019). Development of CAR-T cells for long-term eradication and surveillance of HIV-1 reservoir. *Curr. Opin. Virol.* 38 21–30. 10.1016/j.coviro.2019.04.004 31132749

[B29] Liu B. Zou F. Lu L. Chen C. He D. Zhang X. (2016). Chimeric antigen receptor T cells guided by the single-chain Fv of a broadly neutralizing antibody specifically and effectively eradicate virus reactivated from latency in CD4+ T lymphocytes isolated from HIV-1-infected individuals receiving suppressive combined antiretroviral therapy. *J. Virol.* 90 9712–9724. 10.1128/jvi.00852-16 27535056 PMC5068523

[B30] Liu C. Ma X. Liu B. Chen C. Zhang H. (2015). HIV-1 functional cure: will the dream come true? *BMC Med.* 13:284. 10.1186/s12916-015-0517-y PMC4654816 26588898

[B31] Maldini C. R. Claiborne D. T. Okawa K. Chen T. Dopkin D. L. Shan X. (2020). Dual CD4-based CAR T cells with distinct costimulatory domains mitigate HIV pathogenesis in vivo. *Nat. Med.* 26 1776–1787. 10.1038/s41591-020-1039-5 32868878 PMC9422086

[B32] Maldini C. R. Ellis G. I. Riley J. L. (2018). CAR T cells for infection, autoimmunity and allotransplantation. *Nat. Rev. Immunol.* 18 605–616. 10.1038/s41577-018-0042-2 30046149 PMC6505691

[B33] Masiero S. Del Vecchio C. Gavioli R. Mattiuzzo G. Cusi M. G. Micheli L. (2005). T-cell engineering by a chimeric T-cell receptor with antibody-type specificity for the HIV-1 gp120. *Gene Ther.* 12 299–310. 10.1038/sj.gt.3302413 15496956

[B34] McCoy L. E. Burton D. R. (2017). Identification and specificity of broadly neutralizing antibodies against HIV. *Immunol. Rev.* 275 11–20. 10.1111/imr.12484 28133814 PMC5299474

[B35] Minang J. T. Trivett M. T. Bolton D. L. Trubey C. M. Estes J. D. Li Y. (2010). Distribution, persistence, and efficacy of adoptively transferred central and effector memory-derived autologous simian immunodeficiency virus-specific CD8+ T cell clones in rhesus macaques during acute infection. *J. Immunol.* 184 315–326. 10.4049/jimmunol.0902410 19949091 PMC2797560

[B36] Muul L. M. Tuschong L. M. Soenen S. L. Jagadeesh G. J. Ramsey W. J. Long Z. (2003). Persistence and expression of the adenosine deaminase gene for 12 years and immune reaction to gene transfer components: long-term results of the first clinical gene therapy trial. *Blood* 101 2563–2569. 10.1182/blood-2002-09-2800 12456496

[B37] Nixon C. C. Mavigner M. Silvestri G. Garcia J. V. (2017). In vivo models of human immunodeficiency virus persistence and cure strategies. *J. Infect. Dis.* 215 S142–S151. 28520967 10.1093/infdis/jiw637 PMC5410984

[B38] Okuma K. Tanaka R. Ogura T. Ito M. Kumakura S. Yanaka M. (2008). Interleukin-4-transgenic hu-PBL-SCID mice: a model for the screening of antiviral drugs and immunotherapeutic agents against X4 HIV-1 viruses. *J. Infect. Dis.* 197 134–141. 10.1086/524303 18171296

[B39] Ollerton M. T. Berger E. A. Connick E. Burton G. F. (2020). HIV-1-specific chimeric antigen receptor T cells fail to recognize and eliminate the follicular dendritic cell HIV reservoir in vitro. *J. Virol.* 94:e00190-120. 10.1128/JVI.00190-20 PMC7199392 32161179

[B40] Petrovas C. Casazza J. P. Brenchley J. M. Price D. A. Gostick E. Adams W. C. (2006). PD-1 is a regulator of virus-specific CD8(+) T cell survival in HIV infection. *J. Exp. Med.* 203 2281–2292. 10.1084/jem.20061496 16954372 PMC2118095

[B41] Poles M. A. Boscardin W. J. Elliott J. Taing P. Fuerst M. M. P. McGowan I. (2006). Lack of decay of HIV-1 in gut-associated lymphoid tissue reservoirs in maximally suppressed individuals. *J. Acquir. Immune Defic. Syndr.* 43 65–68. 10.1097/01.qai.0000230524.71717.14 16936559

[B42] Porichis F. Kaufmann D. E. (2011). HIV-specific CD4 T cells and immune control of viral replication. *Curr. Opin. HIV AIDS* 6 174–180. 10.1097/coh.0b013e3283454058 21502921 PMC3265969

[B43] Porichis F. Kaufmann D. E. (2012). Role of PD-1 in HIV pathogenesis and as target for therapy. *Curr. HIV AIDS Rep.* 9 81–90. 10.1007/s11904-011-0106-4 22198819 PMC3731769

[B44] Porichis F. Hart M. G. Zupkosky J. Barblu L. Kwon D. S. McMullen A. (2014). Differential impact of PD-1 and/or interleukin-10 blockade on HIV-1-specific CD4 T cell and antigen-presenting cell functions. *J. Virol.* 88 2508–2518. 10.1128/jvi.02034-13 24352453 PMC3958087

[B45] Rafiq S. Yeku O. O. Jackson H. J. Purdon T. J. van Leeuwen D. G. Drakes D. J. (2018). Targeted delivery of a PD-1-blocking scFv by CAR-T cells enhances anti-tumor efficacy in vivo. *Nat. Biotechnol.* 36 847–856. 10.1038/nbt.4195 30102295 PMC6126939

[B46] Roberts M. R. Qin L. Zhang D. Smith D. H. Tran A. C. Dull T. J. (1994). Targeting of human immunodeficiency virus-infected cells by CD8+ T lymphocytes armed with universal T-cell receptors. *Blood* 84 2878–2889. 10.1182/blood.v84.9.2878.bloodjournal8492878 7949163

[B47] Rosenberg S. A. Restifo N. P. (2015). Adoptive cell transfer as personalized immunotherapy for human cancer. *Science* 348 62–68. 10.1126/science.aaa4967 25838374 PMC6295668

[B48] Ruelas D. S. Greene W. C. (2013). An integrated overview of HIV-1 latency. *Cell* 155 519–529. 10.1016/j.cell.2013.09.044 24243012 PMC4361081

[B49] Sahu G. K. Sango K. Selliah N. Ma Q. Skowron G. Junghans R. P. (2013). Anti-HIV designer T cells progressively eradicate a latently infected cell line by sequentially inducing HIV reactivation then killing the newly gp120-positive cells. *Virology* 446 268–275. 10.1016/j.virol.2013.08.002 24074590 PMC3791854

[B50] Said E. A. Dupuy F. P. Trautmann L. Zhang Y. Shi Y. El-Far M. (2010). Programmed death-1-induced interleukin-10 production by monocytes impairs CD4+ T cell activation during HIV infection. *Nat. Med.* 16 452–459. 10.1038/nm.2106 20208540 PMC4229134

[B51] Scholler J. Brady T. L. Binder-Scholl G. Hwang W. T. Plesa G. Hege K. M. (2012). Decade-long safety and function of retroviral-modified chimeric antigen receptor T cells. *Sci. Transl. Med.* 4:132ra53. 10.1126/scitranslmed.3003761 22553251 PMC4368443

[B52] Shin H. Wherry E. J. (2007). CD8 T cell dysfunction during chronic viral infection. *Curr. Opin. Immunol.* 19 408–415. 10.1016/j.coi.2007.06.004 17656078

[B53] Siliciano J. D. Siliciano R. F. (2014). Recent developments in the search for a cure for HIV-1 infection: targeting the latent reservoir for HIV-1. *J. Allergy Clin. Immunol.* 134 12–19. 10.1016/j.jaci.2014.05.026 25117799

[B54] Sodroski J. Goh W. C. Rosen C. Campbell K. Haseltine W. A. (1986). Role of the HTLV-III/LAV envelope in syncytium formation and cytopathicity. *Nature* 322 470–474. 10.1038/322470a0 3016552

[B55] Teigler J. E. Zelinskyy G. Eller M. A. Bolton D. L. Marovich M. Gordon A. D. (2017). Differential inhibitory receptor expression on T cells delineates functional capacities in chronic viral infection. *J. Virol.* 91:e01263-17. 10.1128/JVI.01263-17 PMC5686724 28904197

[B56] Tian X. Zhang A. Qiu C. Wang W. Yang Y. Qiu C. (2015). The upregulation of LAG-3 on T cells defines a subpopulation with functional exhaustion and correlates with disease progression in HIV-infected subjects. *J. Immunol.* 194 3873–3882. 10.4049/jimmunol.1402176 25780040

[B57] Tran A. C. Zhang D. Byrn R. Roberts M. R. (1995). Chimeric zeta-receptors direct human natural killer (NK) effector function to permit killing of NK-resistant tumor cells and HIV-infected T lymphocytes. *J. Immunol.* 155 1000–1009. 7608531

[B58] Trautmann L. Janbazian L. Chomont N. Said E. A. Gimmig S. Bessette B. (2006). Upregulation of PD-1 expression on HIV-specific CD8+ T cells leads to reversible immune dysfunction. *Nat. Med.* 12 1198–1202. 10.1038/nm1482 16917489

[B59] Velu V. Titanji K. Zhu B. Husain S. Pladevega A. Lai L. (2009). Enhancing SIV-specific immunity in vivo by PD-1 blockade. *Nature* 458 206–210. 10.1038/nature07662 19078956 PMC2753387

[B60] Wagner T. A. (2018). Quarter century of anti-HIV CAR T cells. *Curr. HIV AIDS Rep.* 15 147–154. 10.1007/s11904-018-0388-x 29500712 PMC5884727

[B61] Woo S. R. Turnis M. E. Goldberg M. V. Bankoti J. Selby M. Nirschl C. J. (2012). Immune inhibitory molecules LAG-3 and PD-1 synergistically regulate T-cell function to promote tumoral immune escape. *Cancer Res.* 72 917–927. 10.1158/0008-5472.can-11-1620 22186141 PMC3288154

