# Supplementary figures and images for: HIV-1-Specific CAR-T Cells With Cell-Intrinsic PD-1 Checkpoint Blockade Enhance Anti-HIV Efficacy in vivo
Source: Front Microbiol. 2021 Jul 6;12:684016. doi: 10.3389/fmicb.2021.684016 (PMC8290485; doi:10.3389/fmicb.2021.684016)

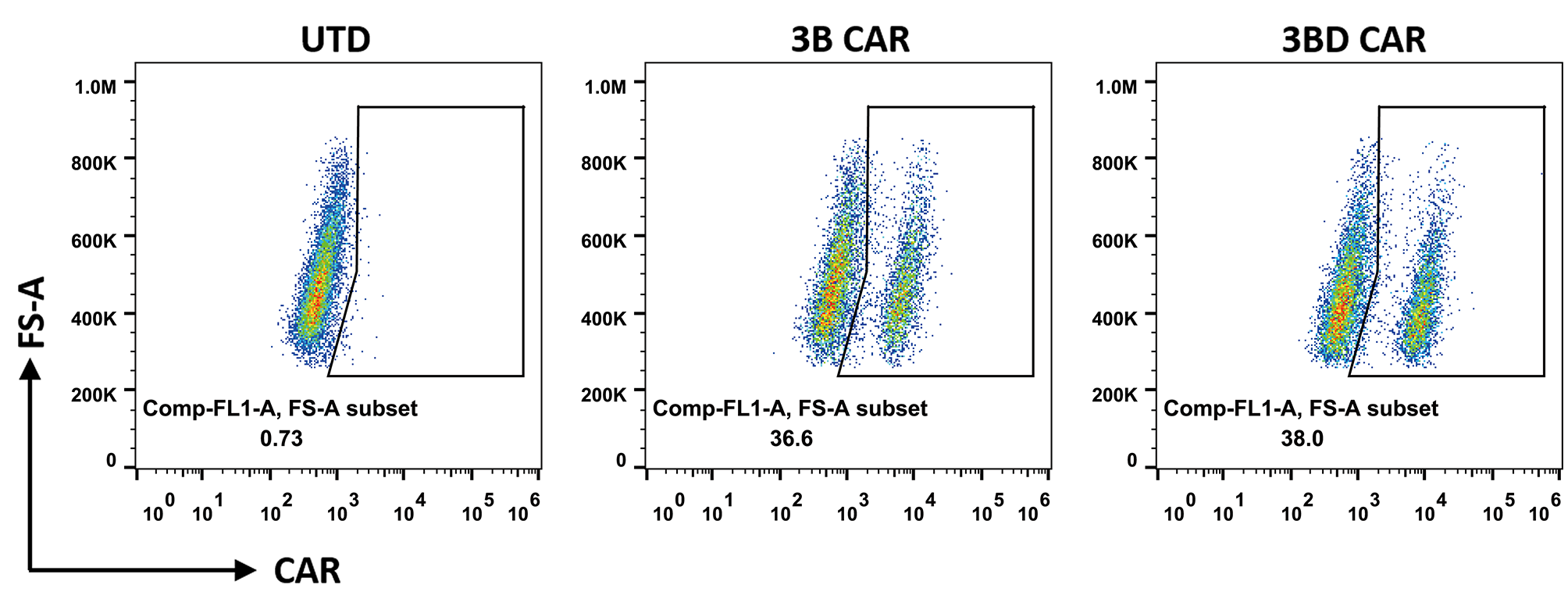

Supplement: Supplementary Figure 1 — Detection of CARs on transduced CD3 T cells before sorting. The 3BNC117 CAR was detected with FITC-labeled goat anti-human IgG antibody; untransduced CD3+ T cells (UTD) served as a negative control. [file Image_1.TIF]

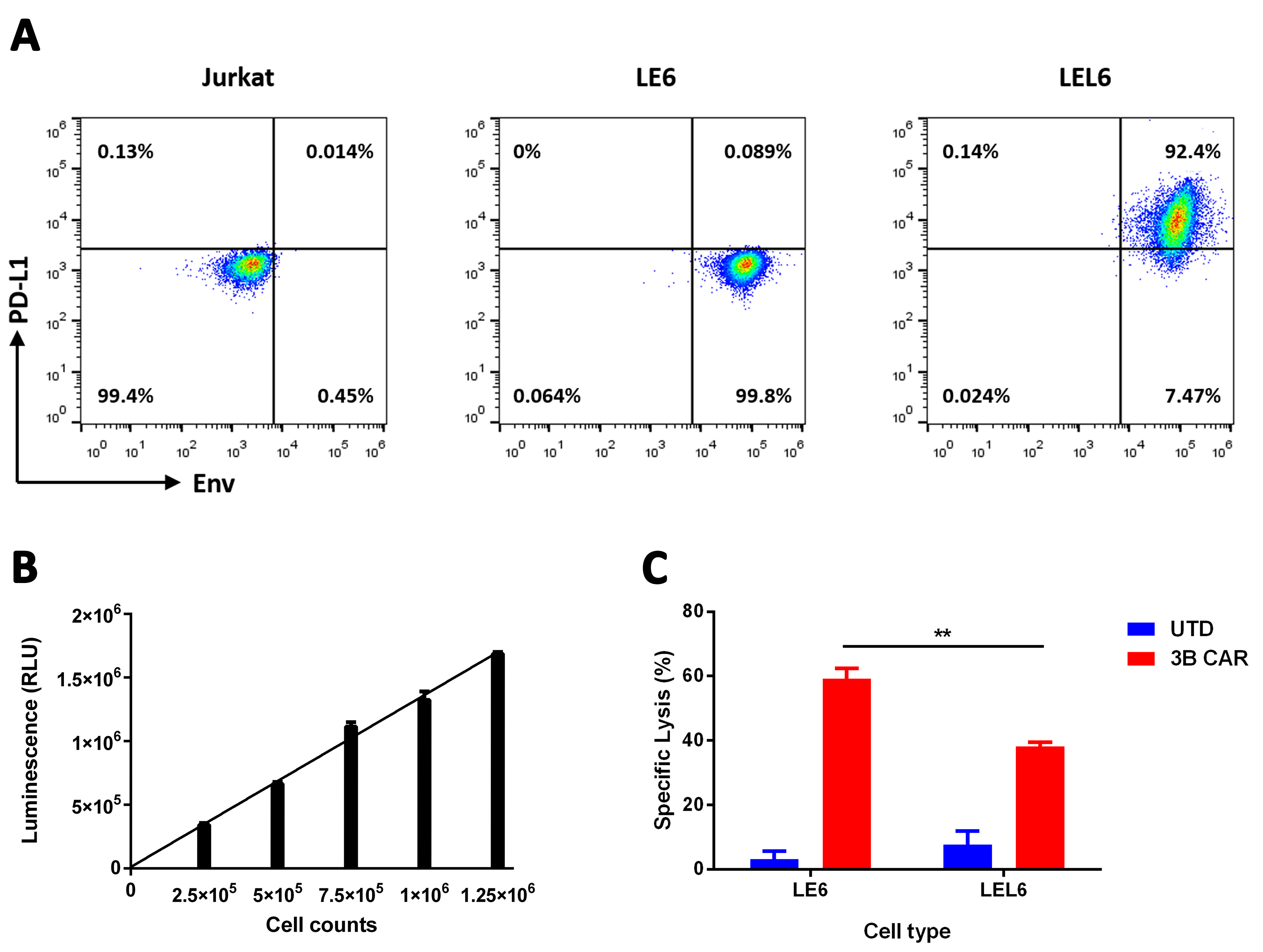

Supplement: Supplementary Figure 2 — Generation of Env-expressing target cells. (A) Detection of Env and PD-L1 on LE6/LEL6 cells. Env was detected with a biotin-labeled goat anti-human gp120 antibody and APC streptavidin. PD-L1 was detected with a PE-labeled mouse anti-human CD274 antibody. Jurkat cells served as a negative control. (B) Detection of luminescence (RLU) on different numbers of LEL6 cells with a Luc assay. (C) PD-L1 inhibits 3B CAR-T cells cytolytic function. 3B CAR-T cells sorted for CAR expression were incubated with LE6 or LEL6 respectively at 10:1 (E: T) ratio for 8 h, and then the direct killing was detected by LDH assay. UTD served as a negative control. Statistical analyses were performed by two-way ANOVA followed by Bonferroni post-test analysis. ∗∗P < 0.01. Data represent the mean ± SEM. [file Image_2.TIF]

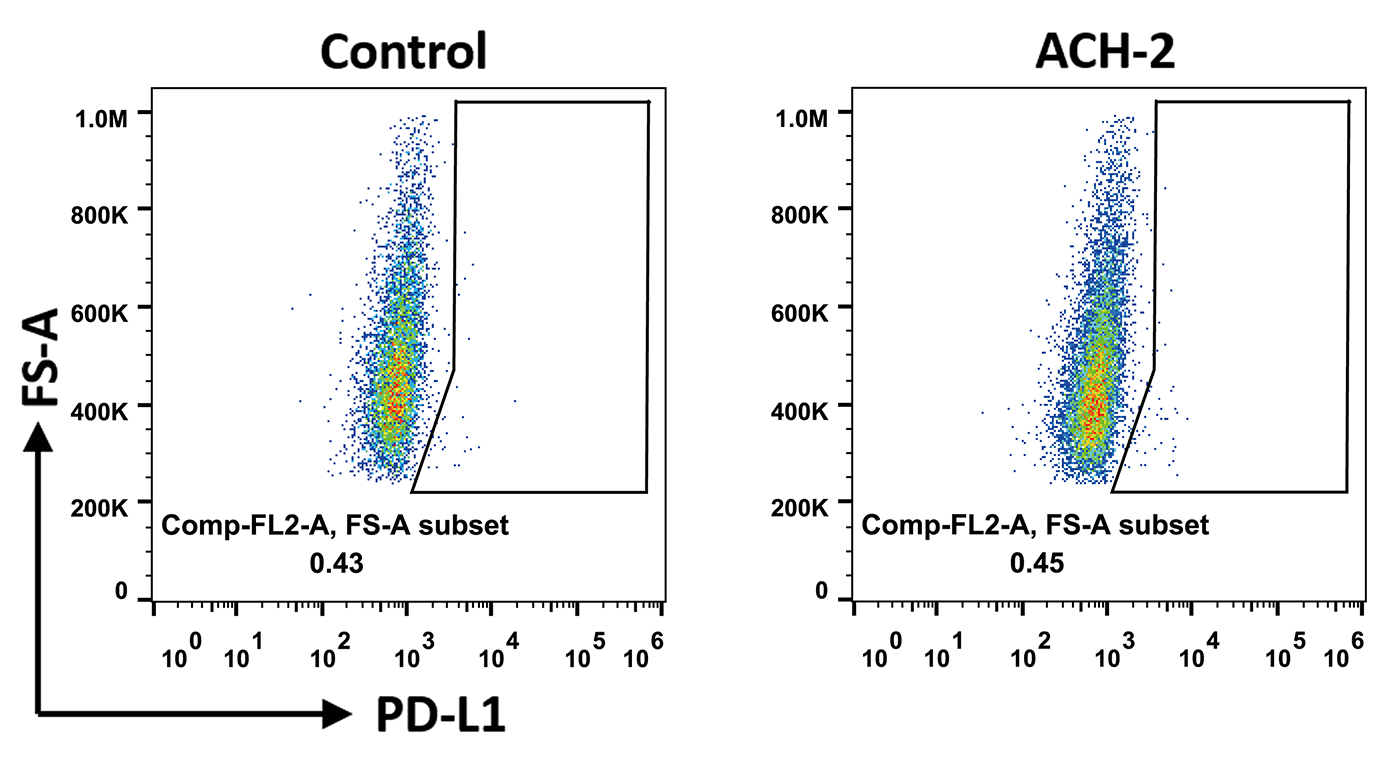

Supplement: Supplementary Figure 3 — Flow cytometry for the detection of PD-L1 in ACH-2 cells. PE-labeled mouse anti-human CD274 antibody was used to determine the expression of PD-L1 on ACH-2 cells. [file Image_3.TIF]
